# Risk of early-onset dementia among persons with tinnitus: a retrospective case–control study

**DOI:** 10.1038/s41598-021-92802-y

**Published:** 2021-06-28

**Authors:** Yen-Fu Cheng, Sudha Xirasagar, Tzong-Han Yang, Chuan-Song Wu, Yi-Wei Kao, Herng-Ching Lin

**Affiliations:** 1 Department of Medical Research, Taipei Veterans General Hospital, Taipei, Taiwan; 2 Department of Otolaryngology-Head and Neck Surgery, Taipei Veterans General Hospital, Taipei, Taiwan; 3 Department of Speech, Language and Audiology, National Taipei University of Nursing and Health Sciences, Taipei, Taiwan; 4 Research Center of Sleep Medicine, College of Medicine, Taipei Medical University, Taipei, Taiwan; 5 Department of Otolaryngology-Head and Neck Surgery, School of Medicine, National Yang-Ming University, Taipei, Taiwan; 6 Department of Health Services Policy and Management, Arnold School of Public Health, University of South Carolina, Columbia, USA; 7 Department of Otolaryngology, Taipei City Hospital, Taipei, Taiwan; 8 College of Science and Engineering, Fu Jen University, New Taipei City, Taiwan; 9 Big Data Research Center, Taipei Medical University, Taipei, Taiwan; 10 Graduate Institute of Business Administration, College of Management, Fu Jen Catholic University, New Taipei City, Taiwan; 11 School of Health Care Administration, Taipei Medical University, 250 Wu-Hsing St., Taipei, 110 Taiwan; 12 Sleep Research Center, Taipei Medical University Hospital, Taipei, Taiwan

**Keywords:** Neurology, Risk factors

## Abstract

Higher rates of poor cognitive performance are known to prevail among persons with tinnitus in all age groups. However, no study has explored the association between tinnitus and early-onset dementia. We hypothesize that tinnitus may precede or occur concurrently with subclinical or early onset dementia in adults younger than 65 years of age. This case–control study used data from the Taiwan National Health Insurance Research Database, identifying 1308 patients with early-onset dementia (dementia diagnosed before 65 years of age) and 1308 matched controls. We used multivariable logistic regressions to estimate odds ratios (ORs) for prior tinnitus among patients with dementia versus controls. Among total 2616 sample participants, the prevalence of prior tinnitus was 18%, 21.5% among cases and 14.5% among controls (*p* < 0.001). Multivariable logistic regression showed and adjusted OR for prior tinnitus of 1.6 for cases versus controls (95% CI: 1.3 ~ 2.0). After adjusting for sociodemographic characteristics and medical co-morbidities, patients with early-onset dementia had a 67% higher likelihood of having prior tinnitus (OR = 1.628; 95% CI = 1.321–2.006). Our findings showed that pre-existing tinnitus was associated with a 68% increased risk of developing early-onset dementia among young and middle-aged adults. The results call for greater awareness of tinnitus as a potential harbinger of future dementia in this population.

## Introduction

Dementia is a disorder characterized by a decline in cognitive abilities involving one or more cognitive domains. The development of dementia involves complex processes involving specific molecular pathways affecting multiple cellular functions of the central nervous system, leading to disruption of the functional networks underlying cognition, behavior and sensorimotor functions, eventually eroding autonomous functioning and decision-making abilities of affected individuals^1,2^. Although the prevalence of dementia increases with age, it may also affect younger individuals.

Early-onset dementia is defined as dementia diagnosed before the age of 65^3,4^ The symptoms of early-onset dementia are similar to those observed among the elderly, behavioral changes, cognitive decline, and psychiatric manifestations. In addition to early-onset adult neurodegenerative disorders such as Alzheimer’s disease, vascular dementia and frontotemporal dementia, early-onset dementia may be caused by delayed onset of childhood neurodegenerative disorders caused by mitochondrial and lysosomal disorders^4,5^. The diagnosis of early-onset dementia is particularly challenging because the current diagnostic criteria require evidence of cognitive impairment and memory loss. Younger patients whose dementia is limited to progressive cognitive decline or focal neurological impairments may not be diagnosed with dementia or receive a delayed diagnosis. Further, studies show that patients with early-onset dementia take a longer time to seek their first consultation for dementia evaluation and for families to become aware of the dementia diagnosis^6^. Considering the newer treatment options that are becoming available to modify the course of dementia, the consequences of delayed or unrecognized early-onset dementia can be serious and unnecessary. Consequences that may be mitigated by early diagnosis include morbidity and stigma suffered by patients, and the economic and resource use burden borne by families and the healthcare system over the subsequent life span.

Tinnitus is a phantom auditory perception in the absence of an objective source of physical sound. Tinnitus is a common and disturbing phenomenon, with reported prevalence rates ranging from 7 to 20% in the general population^7–10^. One study even reported that the incidence of tinnitus was as high as 26.7% for people ages 65–84 years in the United States^11^. Tinnitus can occur due to pathologies occurring at any point between the cochlear apparatus and the auditory cortex. Increasing evidence shows tinnitus to be a disorder involving neuroplastic changes in the central auditory structures that occur when the brain is deprived of its normal input due to cochlear lesions^12^.

Studies show correlation between the presence of poor cognitive performance and tinnitus^13–17^, and a high rate of cognitive impairment is observed among in patients with tinnitus across all age groups^18,19^. Despite these suggestive associations, there are no documented studies that examined associations between tinnitus and early-onset dementia. We hypothesize that tinnitus may precede or occur parallel to subclinical or early dementia among the population younger than 65 years of age. We sought to examine whether tinnitus may represent an early sign preceding early-onset dementia using administrative claims data.

## Methods

This retrospective case–control study used data from the Taiwan National Health Insurance (NHI) Research Database (NHIRD). The NHIRD comprises all medical claims data for approximately 99% of the Taiwanese population (about 24.02 million registered beneficiaries in December 2019) under Taiwan’s NHI program. Many researchers have used the NHIRD to track longitudinal use of medical care and diagnoses over follow-up for research purposes.

The study is based on de-identified administrative data provided by the NHIRD. It was approved and deemed exempt from informed consent requirement by the Institutional Review Board of Taipei Medical University (TMU-JIRB N202005074), and is compliant with the Declaration of Helsinki.

### Study sample

To identify cases, we first identified 206,940 patients with a first-time diagnosis of dementia (ICD-9-CM codes 290.0 ~ 290.4, 294.1, 331.0 ~ 331.2, or 331.82 or ICD-10-CM code F03.90) in an outpatient setting (private clinics or hospital outpatient departments) between January 1, 2010 and December 31, 2016. We included only patients with a documented diagnosis of dementia at least two medical encounters during the study period to improve diagnostic validity. The date of the first-time dementia diagnosis during the study period was assigned as their index date. Next we selected patients aged between 30 and 64 years of age (*n* = 11,361). Finally, we excluded patients with a history of major psychosis or a substance use-related disorder (ICD-9-CM codes 291 ~ 299, 303 ~ 305), stroke (ICD-9-CM codes 430 ~ 438), or traumatic brain injury (TBI) (ICD-9-CM codes 801 ~ 804 or 850 ~ 854) prior to the index date (*n* = 10,053). The reason for excluding patients with a history of TBI was that TBI was reported as a potential risk factor for other neurodegenerative disorders that can be associated with dementia^20^. The remaining 1308 patients with early-onset dementia were included as cases in this study.

To select controls out of the remaining patients, we first excluded those who had ever received a diagnosis of dementia, major psychosis or a substance-related disorder, stroke, or traumatic brain injury, and those aged 65 years or over. We selected one propensity score-matched control per case, matching controls to cases using patient demographic variables (age, sex, monthly income, geographic location and urbanization level of the patient’s residence) and the co-morbidities relevant to dementia development hyperlipidemia, diabetes, coronary heart disease, hypertension, obesity, hearing loss, and alcohol abuse. We matched controls to a corresponding dementia case based on their utilization of any medical service in the index year of the case. For controls, we assigned the date of their first utilization of ambulatory care during the matched year as the index date. A total of 1,308 cases and 1308 controls were analyzed in the study.

### Exposure assessment

Patients with a tinnitus diagnosis were identified based on ICD-9-CM code 388.3. We defined a patient as having tinnitus if they had at least one claim with a diagnosis of tinnitus prior to the index date during the study period.

### Statistical analysis

Statistical analyses were carried out using the SAS system (SAS System for Windows, vers. 9.4, SAS Institute, Cary, NC). Chi-square test and t-tests were performed to examine differences in patient demographics and medical comorbidities between cases and controls. We used multivariable logistic regressions to estimate the odds ratios (ORs) of prior tinnitus among patients with dementia versus controls. We used two-sided *p* < 0.05 for statistical significance.

## Results

Study patients’ mean age was 59.5 years. Table 1 presents the sociodemographic characteristics and comorbidities among cases and controls, showing no significant differences in age, sex, monthly income, hypertension, and hyperlipidemia between cases and controls. However, there were significant differences in geographic region (*p* = 0.002), and the prevalence of diabetes (*p* = 0.047), coronary heart disease (*p* < 0.001) and hearing loss (*p* = 0.002).

**Table 1:** Demographic characteristics and comorbidity status of patients with early-onset dementia and matched control patients in Taiwan (n = 2616).

Variable	Patients with early-onset dementia (*n* = 1308)		Controls (*n* = 1308)		*p* value
	Total no	%	Total no	%	
Age (years), mean (SD)	59.64 (6.49)		59.41 (6.61)		0.369
Males	505	38.6	517	39.5	0.659
**Monthly income**					0.147
< NT$1 ~ 15,841	323	24.7	358	27.4	
NT$15,841 ~ 25,000	544	41.6	550	42	
≥ NT$25,001	441	33.7	400	30.6	
**Geographic region**					0.002
Northern	482	36.9	428	32.7	
Central	314	24	321	24.5	
Eastern	6	0.5	24	1.8	
Southern	506	38.7	535	40.9	
**Urbanization level**					< 0.001
1 (most urbanized)	460	35.2	407	31.1	
2	516	39.4	466	35.6	
3	263	20.1	302	23.1	
4	9	0.7	42	3.2	
5 (least urbanized)	60	4.6	91	7	
Diabetes	381	29.1	429	32.8	0.047
Hypertension	622	47.6	664	50.8	0.109
Coronary heart disease	247	18.9	344	26.3	< 0.001
Hyperlipidemia	583	44.6	601	45.9	0.504
Hearing loss	75	5.7	41	3.1	0.002
Obesity	9	0.7	11	0.8	0.822
Alcohol abuse	3	0.2	0	0	0.248

Table 2 presents the prevalence of prior tinnitus among cases and controls. Among the total sample, the prevalence of prior tinnitus was 18%, 21.5% among cases and 14.5% among controls (*p* < 0.001). Univariable logistic regression analysis showed an unadjusted OR for prior tinnitus of 1.610 among cases relative to controls (95% CI: 1.315 ~ 1.971, *p* < 0.001).

**Table 2:** Prevalence of prior tinnitus crude odds ratio (OR), and 95% confidence interval (CI) for prior tinnitus among early-onset dementia patients and controls.

Presence of prior tinnitus	Total (*n* = 2616)		Patients with early-onset dementia (*n* = 1308)		Controls (*n* = 1308)	
	*n*, %		*n*, %		*n*, %	
Yes	471	18.0	281	21.5	190	14.5
No	2,145	82.0	1,027	78.5	1,118	85.5
OR (95% CI)	–		1.610*** (1.315, 1.971)		1.000	

***p < 0.001.

OR = odds ratio.

Multivariable logistic regression analysis showed that after adjusting for age, income, geographical location, urbanization level, hypertension, diabetes, coronary heart disease, hyperlipidemia, obesity, hearing loss, and alcohol abuse, patients with early-onset dementia were more likely to have had tinnitus before the index date, adjusted odds ratio1.628 (95% CI = 1.321–2.006; *p* < 0.001) (Table 3).

**Table 3:** Multiple regression analysis results showing the adjusted odds ratio (OR) for prior tinnitus of early-onset dementia patients vs. controls (n = 2616).

Variable	Presence of early-onset dementia		
	Adjusted OR	95% CI	*p* value
Prior tinnitus	1.628	1.321–2.006	< 0.001
Male (reference: Female)	0.995	0.846–1.17	0.951
Age	1.003	0.991–1.016	0.596
**Monthly income**			
< NT$15,841 (reference group)	1.000	–	–
NT$15,841 ~ 25,000	1.151	0.943–1.406	0.168
≥ NT$25,001	1.154	0.937–1.422	0.179
**Geographic region**			
Northern (reference group)	1.000	–	–
Central	0.923	0.736–1.158	0.489
Eastern	1.22	0.295–5.033	0.784
Southern	0.911	0.738–1.123	0.383
**Urbanization level**			
1 (reference group)	1.000	–	–
2	1	0.815–1.226	0.998
3	0.783	0.611–1.005	0.055
4	0.165	0.053–0.519	0.002
5	0.568	0.389–0.829	0.003
Hyperlipidemia	1.002	0.848–1.183	0.985
Diabetes	0.896	0.749–1.072	0.231
Hypertension	0.957	0.811–1.132	0.607
Coronary heart disease	0.644	0.533–0.782	< 0.001
Hearing loss	1.781	1.193–2.654	0.005
Obesity	0.787	0.322–1.926	0.600
Alcohol abuse	217,194.037	0– > 9999	0.962

All variables listed in the table were included in the multiple logistic regression model.

Table 4 presents the adjusted odds ratio for prior tinnitus of early-onset dementia patients versus controls stratified by sex, age group and the presence of co-morbidities. There was no statistically significant association between early-onset dementia and interaction terms of age * tinnitus, sex * tinnitus, hypertension * tinnitus, hyperlipidaemia * tinnitus, and hearing loss * tinnitus. In addition, we found that the association of early-onset dementia with prior tinnitus exists regardless whether there was the presence of hypertension, hyperlipidaemia or hearing loss.

**Table 4:** The adjusted odds ratio (OR) for prior tinnitus of early-onset dementia patients vs. controls stratified by sex, age group and the presence of co-morbidities.

Variable	Presence of early-onset dementia		
	Adjusted OR	95% CI	*p* value
**Sex**			0.135*
Males	1.298	0.911–1.849	0.148
Females	1.800	1.385–2.341	< 0.001
**Age**			0.664*
< 45 years old	0.957	0.206–4.448	0.956
≥ 45 years old	1.658	1.340–2.051	< 0.001
**Hyperlipidemia**			0.688*
Yes	1.673	1.247–2.244	0.001
No	1.591	1.178–2.149	0.002
**Diabetes**			0.001*
Yes	0.903	0.613–1.331	0.606
No	2.082	1.614–2.685	< 0.001
**Hypertension**			0.404*
Yes	1.512	1.127–2.027	0.006
No	1.827	1.345–2.482	< 0.001
**Coronary heart disease**			0.291*
Yes	1.428	0.945–2.158	0.091
No	1.746	1.364–2.236	< 0.001
**Hearing loss**			0.289*
Yes	3.004	1.051–8.601	0.040
No	1.563	1.262–1.938	< 0.001

*Denotes *p* value for interaction term.

## Discussion

To our knowledge, this study may represent the first population-based retrospective study to explore a possible association between tinnitus and subsequent early-onset dementia. We found that pre-existing tinnitus was significantly associated with dementia occurrence in the population aged 30–64 years of age, Tinnitus was associated with a 63% higher risk of early-onset dementia.

Dementia is generally regarded as a multifactorial disease, and its incidence increases with age. Several pathologies have been observed to contribute to the development of dementia, including neurodegenerative proteinopathies, vascular disease, dysregulated inflammation, etc. There is usually a considerable delay in the diagnosis of dementia, especially for early-onset dementia, which is estimated to take at least 2–4.4 years after the first onset of its various symptoms^21–23^. The delays may arise out of low prevalence causing a low index of suspicion among younger age groups, the large variety of etiologies, and confounding with neuropsychiatric symptoms and consequent misdiagnosis of early-onset dementia. Subtle neuro-pathologic changes usually precede a definitive diagnosis of dementia. If the associated neuropathology also involves the neural circuitry that triggers tinnitus, it appears plausible that tinnitus may precede or coexist with clinically detectable dementia symptoms such as impairment of memory, early signs of deterioration of executive functions, and impairments in visuoconstructional/ perceptual-motor functions, language functions, and social cognition which typically manifest in the later stages of dementia.

Cognitive impairment has been reported as a common occurrence among tinnitus patients^13–17,24^. Mild cognitive impairments (MCI), an intermediate state before dementia patients transition into clinically evident dementia, has been reported among patients with tinnitus. Other studies have shown that tinnitus is associated with cognitive deficits, and that tinnitus patients on the severe end of spectrum are at high risk of serous cognitive deficits^18,19^. However, the causal mechanism that links tinnitus and dementia remain elusive.

Both tinnitus and dementia represent clinical manifestations of heterogeneous pathologies involving complex neurological and functional processes. Tinnitus is linked to dysregulated neural synchrony across neural ensembles along the auditory pathway^25^. Accumulating evidence shows that tinnitus may occur concurrent with structural and functional disruptions of a diverse range of neuro-sensory structures, ranging from the peripheral and central auditory pathways to areas of the brain that are unrelated to normal hearing and processing of auditory stimuli. Animal and human neuroimaging studies have revealed neural tissue changes similar to those observed in tinnitus-associated areas of the brain in unrelated areas including those associated with cognition impairment and/or dementia, including the ventromedial prefrontal cortex^26^, parietal cortex^27^, anterior cingulate cortex^13,17,28^, prefrontal cortex^29^, amygdala^17,30^, hippocampus^13,17,30^, nucleus accumbens^31^, insula^13,17^, and thalamus^31^. Imaging studies have shown pathological changes in the hippocampus, amygdala and prefrontal cortex in the preclinical phase of dementia^32–37^. Autopsy studies of patients with tinnitus and cognitively normal brain function before death also show proteinopathy or accumulation of abnormal protein aggregates in the brain areas related to tinnitus^38^. These results suggest a shared neuronal pathology between tinnitus and dementia, and support the hypothesis that tinnitus may precede or occur concurrent with subclinical or early-onset dementia. One research implication of our study finding and the related hypothesis is to look for evidence of abnormal protein aggregates in tinnitus patients with normal cognition to explore the potential role of dementia-associated proteinopathy in the pathogenesis of tinnitus.

Our study may be the first and largest population-based study to examine the association between tinnitus and dementia in patients aged under 65 years. A key strength of the study is the ability to pool a large number of early-onset dementia cases from a nationwide medical care dataset. Another strength is the universal access, national health insurance system source of the data, enabling access to uninterrupted follow-up data of the entire population with comprehensive longitudinal data on comorbidities and demographic characteristics.

There are still some study limitations. First, an epidemiological association by itself does not imply biological causality. Our finding suggests a potential link between tinnitus and early-onset dementia which may serve as an early warning sign to elevate awareness of dementia risk among tinnitus patients to proactively watch for the early signs of dementia among young and middle-aged patients. Second, because of data privacy and confidentiality under the Personal Data Protection Law and related regulations of the NHIRD, it was not possible to validate or supplement the claims data by direct patient contact. Therefore, critical items of data on the risk factors for dementia and the severity of tinnitus that are not documented in medical claims could not be obtained. These data include, such as family history, laboratory data, genetic test data (such as *Apolipoprotein E*), severity of dementia, imaging test results, hearing test results, and tinnitus severity scores. Further several known confounders involved in the development of dementia were not accounted for in the analyses, including smoking, educational level, occupation, specific environmental features obesity, hearing loss, and alcohol intake. To mitigate selection bias, we used the available data on the factors known to influence dementia occurrence to select propensity score–matched control patients. Third, this study used a case–control method which did not allow to establish causation due to its retrospective nature. Finally, the present study found that coronary heart disease was negatively associated with early dementia while no significant associations of early dementia with hypertension, diabetes or hyperlipidaemia were observed. We speculate that the medications such as statins or aspirin for the treatment of coronary heart disease could be one explanation for low odds of early-onset dementia on patients with coronary heart disease. Further studies are encouraged to explore the relationship between statins or aspirin and early-onset dementia.

## Conclusion

Our findings showed that pre-existing tinnitus is associated with a 1.675-fold increase in the risk of early-onset dementia among the young and middle-aged population. Additional studies in other populations are encouraged to confirm the relationship between early-onset dementia and tinnitus and to explore the possible mechanisms behind this relationship. Further studies are also needed to clarify the shared underlying pathophysiology between tinnitus and dementia, and to explore whether early detection and treatment of tinnitus may prevent or delay early onset dementia.
